# Perceived Efficiency Outcomes, Sources and Awareness of Online Health Information among the Elderly during COVID-19

**DOI:** 10.3390/ijerph18158121

**Published:** 2021-07-31

**Authors:** Gizell Green, Riki Tesler, Cochava Sharon

**Affiliations:** 1 Nursing Department, School of Health Sciences, Ariel University, Ariel 40700, Israel; cochishar@gmail.com; 2 Department of Health Systems Management, School of Health Sciences, Ariel University, Ariel 40700, Israel; rikite@ariel.ac.il

**Keywords:** online health information, elderly, COVID-19, perceived efficiency, awareness of online sources

## Abstract

The Internet and social media are crucial platforms for health information. Factors such as the efficiency of online health information, the outcomes of seeking online health information and the awareness of reliable sources have become increasingly important for the elderly during the COVID-19 pandemic. This study aimed to examine differences between elderly individuals’ income above and below the average monthly wage in relation to their online health information efficiency and the outcomes of seeking online health information; to evaluate types of online information sources with online health information efficiency and the outcomes of seeking online health information; and to explore online health information efficiency as a mediator between health status and awareness of online sources. A cross-sectional study design was conducted with 336 elderly participants age 65 or older. The participants volunteered to complete a questionnaire. No differences were found between the two groups regarding efficiency in retrieving health information from official online health sites and Google. Perceived efficiency mediated health status and awareness of online sources. In these challenging times, it is important to provide a tailor-made education strategy plan for reliable sources of online health information for the elderly, in order to enhance their technology safety skills. It is also important to explore other mediating variables between health status and awareness of online sources.

## 1. Introduction

The Internet and social media platforms are crucial venues for health information [1,2], which in this study refers to all aspects of personal health, including physical and mental health handled in either physical or digital form [3]. The Internet and social media have become common channels for people to search for information about health, nutrition and everyday life [2,4,5]. eHealth literacy is the ability of individuals to use information and communication technologies such as the Internet to enhance health care [6].

Researchers have found that eHealth literacy is related to improved subjective health status [7,8,9], quality of life [10,11] and low risk of chronic illness [6]. eHealth literacy has been found to be a benefit factor for individuals aged 50–60 years [12]. In a study that developed an eHealth literacy scale, it was found that the awareness of different online health sources and perceived online health efficiency dimensions of the scale were the most developed dimensions, as users scored significantly higher in these two dimensions as compared to the others [13]. Therefore, in the current study, we focused on these two dimensions. Furthermore, online health information, which in this study refers to all aspects of health including physical and mental health handled in online form [3], can relate to other factors, such as the individual’s income. Individuals with lower education and income levels, as well as those who are in poor health, have a higher risk of misinterpreting health information [14]. It is important to note that different sources of health information on the Internet are becoming ever more diverse and these different sources can impact healthcare decision making. Furthermore, little is known about the reasons influencing people’s choice of online health sources [11].

The Internet offers sources of online health information, but the reliability of the data is often questionable. Uncertainties regarding the quality of online health information might have a harmful influence on individuals’ health-related decisions [15]. Individuals with high education levels and incomes tend to find health information on hospital websites, which are considered a reliable source [16]. Additional research demonstrated concerns related to social media (which may be considered an unreliable source) when used as a health information source [17]. Another source that is very common for seeking online health information is the Google search engine (which may also be considered an unreliable source). Occasionally, Google’s quality of online health data can be untrustworthy. It is known that the quality of online health data may have a harmful effect on individuals’ health-related decisions [15]. Therefore, it is important to investigate the relationship between different sources of online health information according to their reliability measure, the perceived efficiency and the perceived outcomes.

Moreover, the lack of awareness of online sources can create avoiding behavior and is one of the reasons for not using devices to access digital health [18]. It is becoming more complicated for the elderly to be able to access reliable health information. The Internet’s advantage can be used in a smart and careful way, especially during the COVID-19 pandemic, in order to enhance patients’ health status.

Although internet use among the elderly (age 65 or older) is growing, this population remains helpless due to the continuously increasing technological strains [19]. A study among elderly women participants who were recently diagnosed with breast cancer found that about half were categorized as having limited eHealth literacy [20]. Another study found that older men (age 65 or older) who were non-native English speakers (the dominant language on the Internet) had low levels of eHealth literacy [21]. These concerns are emphasized because of the global COVID-19 pandemic.

A worldwide pandemic broke out in 2019, called COVID-19 [22,23]. The COVID-19 pandemic has affected the quality of life of people worldwide. Physical isolation has been advised, which has strongly affected people’s lives [24], particularly those in at-risk groups, such as individuals 65 years of age and older. With the new restrictions imposed globally, the Internet has become the main venue for all aspects of life and health. Some of the main resources that the Internet provides are knowledge and health services for patients. Therefore, factors such as the perceived efficiency of online health information (referring to the effectiveness in the search for and use of online health information [13]), the outcomes of seeking online health information and the awareness of reliable sources (referring to the recognition of trustworthy online health resources [13]) have become more important than ever [7,8,9,10,25,26].

### Research Aims

This study has three aims: first, to examine differences between elderly individuals who earn above average monthly wage and those who earn below average monthly wage in relation to their online health information efficiency and the outcomes of seeking online health information during the COVID-19 pandemic; second, to evaluate types of online information sources used by the elderly, their perception of online health information efficiency and the outcomes of seeking health information during the COVID-19 pandemic; and third, to explore a model of online health information efficiency as a mediator between health status and awareness of online sources during the COVID-19 pandemic.

## 2. Materials and Methods

### 2.1. Study Design

A cross-sectional study design with a convenience sample was performed and reported according to the EQUATOR and STROBE checklists for cross-sectional studies [27]. The data were collected from May 2020 to August 2020.

### 2.2. Participants and Procedure

The study participants consisted of 336 elderly people aged 65 or older throughout Israel. We chose this population for three reasons: First, people over 65 years of age face more health challenges, making them a more vulnerable group. Second, the channels to obtain health information have been reduced and the main channel is online due to social distancing recommended during the COVID-19 pandemic. Third, due to information technology developments, it might difficult for the elderly to identify reliable online health information [21,22]. As of the beginning of May 2020, the number of total COVID-19 cases (people who were infected with the virus) in Israel was 16,101 with 2225 deaths (Worldometer, 2021) [28]. Participants were enrolled and data were collected through iPanel (https://www.ipanel.co.il/en/ (accessed on 31 May 2021)), which is an online sampling service that allows for fast responses, striving for a representative sample based on the population’s sociodemographic characteristics, such as gender, age and health status. This panel is the largest panel survey in Israel and holds high quality research codes from the European Society for Opinion and Marketing Research (ESOMAR) [29,30].

An introductory e-mail was sent to potential participants via the iPanel database system. The e-mail directed participants to read the research objectives in detail as well as their rights, such as the right to withdraw at any time from the research, and included the contact details of the researchers. Then, they had the option to electronically sign informed consent to participate in the research and complete the questionnaire if they were interested, which took 10 min to complete. The survey directions emphasized its anonymity and confidentiality. The e-mail was sent to 1597 potential candidates and 336 expressed interest and participated in the study. The response rate for study participation was 27%, which was considered a good response rate.

### 2.3. Measures

To evaluate participants’ perceived efficiency of online health information, awareness of online sources, perceived outcomes of seeking online health information and health status, the questionnaire was composed of four sections. The first section referred to sociodemographic and background characteristics, which gathered information on participants’ age, health status and sources from which they derived their health information. A list of online sources from which to choose was provided to the participants, including sources considered as reliable (e.g., official hospital sites) and unreliable (e.g., social networks).

The second section referred to the perceived efficiency of the online health information and awareness of different types of online sources. There were two subcategories from the eHealth literacy scale: (A) The perceived efficiency of online health information. This subcategory contained 4 items, including the following statement: “I feel confident using information from the Internet to make successful health decisions.” The internal consistency of this subcategory was 0.76 (Cronbach’s alpha). (B) Awareness of online sources. This subcategory consisted of 3 items, including: “I know where to find helpful health resources on the Internet.” The internal consistency was 0.85 (Cronbach’s alpha). Answers were based on a 5-point Likert-like scale ranging from 1 (“completely disagree”) to 5 (“completely agree”) [13].

The third section referred to perceived outcomes of seeking online health information and consisted of 8 items, including: “I updated my knowledge regarding health innovations.” The internal consistency of this subcategory was 0.91 (Cronbach’s alpha). The scale used a 5-point Likert-like scale ranging from 1 (“completely disagree”) to 5 (“completely agree”) [31].

Numerous researchers fluent in English with specialty in eHealth literacy checked the reliability of the translation of the scale from English into Hebrew and vice versa.

### 2.4. Data Analysis

Statistical analysis was performed using two types of software. The first type was the Statistical Package for the Social Sciences (SPSS TM) version 21.0 (IBM, Chicago, IL, USA) and was performed as follows: Cronbach’s alpha, descriptive statistics, t-tests and one-way ANOVA. The second type, the Process, was developed to examine the mediation model [32].

### 2.5. Ethical Considerations

The study protocol was approved by the Ethics Committee of the Ariel University, confirmation number: AU-HEA-GG-20200329-1. The University Institutional Review Board gave permission to conduct this research. Participants were voluntarily recruited and informed of the goals of the research. They also signed an informed consent form before answering the questionnaire. The volunteers were assured that they had the right to withdraw from the research at any time, that their answers would be kept confidential and that the questionnaires would be analyzed anonymously.

## 3. Results

The mean age of the participants was 68 years (SD = 2.91). For frequency and percentage of background characteristics, see Table 1.

Most of the participants were female, married, Jewish and had a high school education level and had income above average wage.

To examine the first research aim (differences between elderly individuals’ income above compared to below average monthly wage regarding online health information efficiency and outcomes), we performed an independent t-test analysis (Table 2).

There were no differences between the two groups as to their online health information efficiency or perceived outcomes of seeking online health information.

The results of the examination of the second research aim (evaluating types of online information sources, the perception of online health information efficiency and their outcomes) are described in Table 3.

When comparing the reliability of different online sources, health information from official health sites (such as hospitals) and Google were more efficient than those who retrieved health information from social networks (*p* < 0.01 and *p* < 0.05, respectively). However, there were no differences between the two groups regarding retrieved health information from official online health sites (such as hospital sites) and Google as to efficiency (*p* > 0.05). In addition, the group that retrieved health information from official online health websites perceived the outcomes of seeking online health information more positively than the group that retrieved health information from social networks (*p* < 0.05). However, there were no differences between the two groups regarding retrieved health information from official online health sites (such as hospital sites) and Google as to perceived outcomes of seeking online health.

In order to examine the relationship between research variables, we conducted a Pearson correlation analysis (Table 4).

There were weak relationships between health status, awareness of online sources and perceived efficiency (*r* = 0.14, *p* < 0.05; *r* = 0.19, *p* < 0.00), respectively. Significant medium up to strong relationships were found between awareness of online sources, perceived efficiency and perceived outcomes of seeking online health information (r = 0.62, *p* < 0.00; r = 0.56, *p* < 0.005), respectively. Moreover, relationships were found between perceived efficiency and perceived outcomes of seeking online health information (r = 0.67, *p* < 0.00).

**Figure 1:**
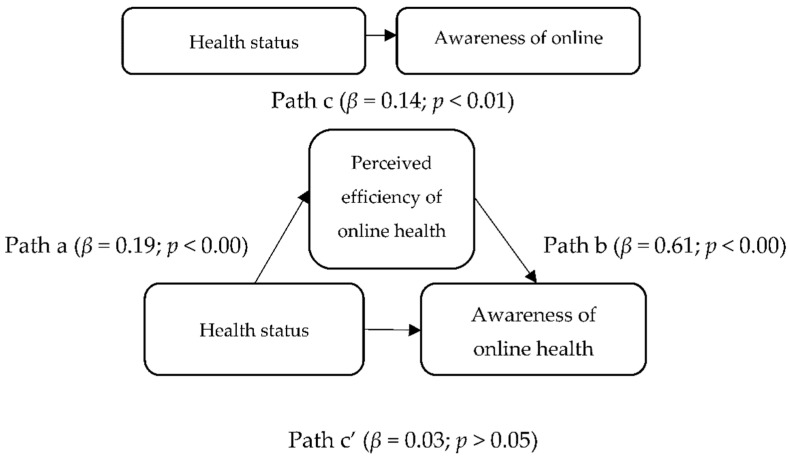
Mediation model.

In order to examine the third research aim (exploring a model of online health information efficiency as a mediator between health status and awareness of online sources), we conducted a series of regression analyses via PROCESS analysis [33] (Figure 1).

Figure 1 shows the relationship between health status, awareness of online sources and perceived efficiency, with perceived efficiency as the mediation variable. In step 1, the regression of health status regarding awareness of online sources was significant (β = 0.14, *p* < 0.00). Step 2 shows that health status as perceived efficiency mediator was also significant (β = 0.19, *p* < 0.00). Step 3 shows that regression of the mediator (perceived efficiency) with awareness of online sources controlling health status was also significant (β = 0.61, *p* < 0.00). Step 4 of the analysis revealed that when controlling for the mediator (perceived efficiency), health status was not a significant predictor of awareness of online sources (β = 0.03, *p* = 0.62). In addition, health status had a positive indirect effect on awareness of online sources through perceived efficiency (β = 0.12, SE = 0.03, 95% CI [0.05, 0.18]). Accordingly, it was found that subjective perceived efficiency fully mediated the relationship between health status and awareness of online sources.

## 4. Discussion

The study strived to (a) examine differences between elderly individuals’ income above and below the average monthly wage in relation to their online health information efficiency and the outcomes of seeking online health information during the COVID-19 pandemic period; (b) evaluate types of online information sources used by the elderly, their perception of online health information efficiency and the outcomes of seeking online health information; and (c) explore online health information efficiency as a mediator between health status and awareness of online sources.

First, no differences were found between the elderly population’s income above and below the average monthly wage in relation to their online health information efficiency and the outcomes of seeking online health information. Surprisingly, the research literature demonstrated inconsistency in this respect. One study found that among the elderly with a low income, lower levels of internet usage were attributable to the absence of financial funds for purchasing technological assistance [33]. Moreover, the elderly with lower income were associated with the use of internet activities such as finding health information, cooperating with health care workers and assessing health [33,34,35]. A possible explanation for our surprising results is that seeking online health information is related to many reasons and is a combination of eHealth literacy, phone possession, technical ability, as well as social recognition of health requirements [18], highlighting the large-scale use of mobile phones to promote health, described as “online health lifestyle” [36]. Another possible explanation is that although the elderly appears to trust persons with whom they are able to actively discuss their health, as opposed to accessing an inanimate source as the Internet [37], the Internet has become a main and important technological channel for obtaining health information for this population while we are in the midst of a global pandemic with the requirement of social distancing and the accessibility of smart phones at convenient prices. Therefore, we conclude that in these challenging times, it is important for government and health care organizations to provide appropriate eLiteracy evaluation and to provide an education plan for the elderly so that they can enhance their technology and safety skills.

Second, we found that the groups that retrieved health information from official online health sites considered their efficiency to be higher than the group that retrieved health information from social networks. In addition, the group that retrieved health information from official online health sites perceived outcomes of seeking online health information more positively than the group that retrieved health information from social networks. Similarly, we found that hospital/institutional websites (considered as reliable sources) were the most popular internet sites used and it was of the essence that hospitals made critical information easy to find and understand [16].

One concern emerged from the results: the group that retrieved health information from Google considered its efficiency to be higher than the group that retrieved health information from social networks. Similar to our results, one study found that age was one of the factors that could explain differences in the capability of examining the value of information [7]. Another study found that younger participants were more confident in distinguishing reliable or unreliable facts of health-related internet information [19]. It is essential to know where to find reliable health online sources to develop electronic health abilities [7]. In this study, perhaps because Google has great popularity including among the elderly, it was mistakenly considered a reliable health information source. Therefore, examining accessibility of reliable online sources and tools of health information is important for the elderly [37].

Third, it was found that perceived efficiency fully mediated the relationship between health status and awareness of online sources. Health status has been extensively researched in many aspects of internet health information [11,38,39]. In addition, health status and internet use affect the frequency and amount of online health information that is sought [7,40]. After a broad review of contemporary research literature, we did not find research that performed in-depth analysis for detecting online health information efficiency as mediator between health status and awareness of online sources. The novelty in the present study is the finding that the health status of an elderly person predicts their awareness of sources of online health information only when perceived efficiency of this health information serves as a full mediator. When controlling the mediating variable, perceived efficiency, the relation between health status and awareness of sources for online health information is canceled. Our alternative explanation for this important finding is due to the recurring lockdowns and extensive recommendations for social distancing for the elderly population in COVID-19 times, causing health and medicine in all aspects to be consumed almost exclusively via the Internet and so the perceived efficiency of the Internet for health needs has become a very significant and influential mediator.

## 5. Conclusions

First, no differences were found between elderly individuals’ average monthly wage and their online health information efficiency and seeking online health information. These two groups perceive their online health information efficiency and seeking online health information equally. Therefore, the health care system needs to provide guidance in finding trustworthy online health information oriented to elderly populations since it has become accessible to everyone due to affordably priced technology devices with internet access.

Second, there were no differences found between the two groups regarding retrieved health information from official online health sites (such as hospital sites) and Google as to the efficiency of health information and perceived outcomes of seeking online health. Moreover, the group that retrieved health information from Google considered its efficiency to be higher than the group that retrieved health information from social networks. The elderly mistakenly consider Google to be a reliable source of online health information. The health care system needs, especially during the COVID-19 period, to provide a tailor-made education strategy plan for distinguishing between unreliable and reliable sources of online health information [2] for the elderly, in order to enhance their technology safety skills.

Third, perceived efficiency fully mediated the relationship between health status and awareness of online sources. We conclude that perceived efficiency has a crucial mediating role between these two variables in relation to the elderly. Therefore, in future research it is necessary to explore this model while adding other mediating variables in order to fully understand the relationship between those variables. Today, gaining deep understanding of online health information among the elderly is not a luxury, but a vital necessity, especially since they are increasingly dependent on the online health information.

### Limitations

There are several limitations to this study. First, our findings are based on a self-reporting questionnaire and not an objective tool. More studies, measuring the actual use of online health information searches, are required.

Second, the cross-section study design with convenience sampling approach limits generalizability of the study results and the representativeness of the study sample. Therefore, it is required to evaluate online health information in a large sample and in different countries. The third limitation is the use of one tool (the questionnaire) in this study. Therefore, future studies should focus on and use various tools, such as observations or semi-structured interviews, which might be useful for detecting more complex and deeper insights and explanations for measuring online health information search behavior phenomena.

## Figures and Tables

**Table 1:** Background characteristics of the study population.

Background Characteristic		*n* = 336	
		*n*	%
Gender	Male	152	45
	Female	184	55
Status	Single	6	2
	Married	256	76
	Divorced	46	14
	Widowed	28	8
Religious	Jewish	333	99
	Muslim	3	1
Religiosity	Secular	226	67
	Traditional	74	22
	Religious	30	9
	Very religious	5	2
Education	High school	74	22
	Diploma	114	34
	Bachelor’s degree	100	30
	Master’s degree	48	15
Financial Status	Above average	179	53
	Below average	149	44

Abbreviations: SD, standard deviation.

**Table 2:** Differences between elderly individuals’ income above and below average monthly wage regarding online health information efficiency and outcomes.

	Elderly Individuals’ Income above the Average Monthly Wage (*n* = 149)		Elderly Individuals’ Income below the Average Monthly Wage (*n* = 179)		t	* *p*
Variable	Mean	SD	Mean	SD		
Perceived efficiency of online health information	3.51	0.77	3.64	0.81	1.63	0.10
Perceived outcomes of seeking health information	3.73	0.81	3.85	0.84	1.26	0.21

Notes: * *p* < 0.05; Abbreviations: SD, standard deviation.

**Table 3:** Types of online sources used by the elderly, their perception of online health information efficiency and the outcomes of seeking online health information.

	Social Networks (Unreliable)		Official Online Health Sites (Reliable) (*n* = 179)		Google (Unreliable) (*n* = 149)		F	* *p*
Variables	Mean	SD	Mean	SD	Mean	SD		
Online health information efficiency	3.10	0.80	3.66	0.78	3.56	0.73	4.44	0.01
Perceived outcomes of seeking online health information	3.64	0.88	3.87	0.83	3.79	0.79	3.27	0.04

Notes: * *p* < 0.05; Abbreviations: SD, standard deviation.

**Table 4:** Pearson correlations between the variables.

Variables	Health Status	Awareness of Online Sources	Perceived Efficiency	Perceived Outcomes of Seeking Online Health Information
Health status	1	0.14 *	0.19 **	0.09
Awareness of online sources		1	0.62 **	0.56 **
Perceived efficiency			1	0.67 **
Perceived outcomes of seeking online health information				1

Notes: * *p* < 0.05; ** *p* < 0.00.
